# COVID-19 unfolding filariasis: The first case of SARS-CoV-2 and *Wuchereria bancrofti* coinfection

**DOI:** 10.1371/journal.pntd.0008853

**Published:** 2020-11-09

**Authors:** Mouhand F. H. Mohamed, Sara F. Mohamed, Zohaib Yousaf, Samah Kohla, Faraj Howady, Yahia Imam

**Affiliations:** 1 Medicine Department, Hamad Medical Corporation, Doha, Qatar; 2 Department of Laboratory Medicine and Pathology, Hematology Section, Hamad Medical Corporation, Doha, Qatar; University of Utah, UNITED STATES

## Abstract

With the evolution of the Coronavirus Disease 2019 (COVID-19) pandemic, the number of patients brought to medical attention has increased. This has led to the unmasking of many coexisting occult infections and comorbidities such as tuberculosis, dengue, human immunodeficiency viral infection, diabetes, and hypertension. We report the first case of Severe Acute Respiratory Syndrome Coronavirus 2 (SARS-CoV-2) infection, unveiling the diagnosis of asymptomatic filariasis. A 37-year-old gentleman presented with shortness of breath, fever, and cough. He was found to have COVID-19 pneumonia. During his stay, microfilaria of *Wuchereria bancrofti* was detected incidentally on a blood smear exam. Consequently, the patient received appropriate treatment for both conditions. In order not to miss relevant concomitant diagnoses, it is prudent to keep a broad differential diagnosis when faced with SARS-CoV-2–infected patients; this is especially true when atypical symptoms are present or in areas endemic with other infections.

**Key learning points**

- Severe Acute Respiratory Syndrome Coronavirus 2 (SARS-CoV-2) coinfection with other microorganisms has been reported.
- We reported the first case of filariasis and SARS-CoV-2 coinfection.
- This coinfection is likely underreported or overlooked in endemic areas.
- Complex immunological interaction influence the clinical presentation of SARS-CoV-2 and filariasis.
- Clinicians must maintain a broad differential diagnosis in endemic areas or the setting of atypical SARS-CoV-2 infection.

## Introduction

Filariasis is an infectious tropical disease that poses a significant health challenge [1]. It is common in the tropics and subtropics of Asia, Africa, the Western Pacific, and parts of the Caribbean and South America [2]. According to the Centers for Disease Control and Prevention (CDC), over a hundred million people have lymphatic filariasis. Nematodes, including *Brugia malayi* and *Brugia timori*, cause the disease, but *Wuchereria bancrofti* is the most common.

The diagnosis is challenging because microfilaria appears periodically in the blood; additionally, its incubation period usually exceeds 6 months [2,3] Incidental detection of microfilaria in the blood, the lymph nodes, and other body tissues and fluids has been reported [4]. Here, we present the first case of filariasis and Severe Acute Respiratory Syndrome Coronavirus 2 (SARS-CoV-2) coinfection.

### Case presentation

A 37-year-old south Asian gentleman with a history of diabetes mellitus (DM) and hypertension (HTN) presented to the emergency unit with a 10-day history of fever, dyspnea, sore throat, cough, nausea, vomiting, and diarrhea. A systemic inquiry revealed a history of chronic mild atypical chest pain that was labeled as musculoskeletal related after extensive workup. The patient is a nonsmoker, consumes alcohol occasionally, and lives in a shared room.

His blood pressure was 118/82 mmHg, the temperature was 39.4°C, heart rate was 118 beats per minute, respiratory rate was 26 breath per minute, and oxygen saturation was 98% on room air. Systemic examination was only remarkable for bilateral lung basal crackles. The initial laboratory investigations were significant for a mild C-reactive protein (CRP) rise (Table 1), and his chest X-ray revealed faint hazy bilateral infiltrates. The SARS CoV-2 infection was confirmed by a nasopharyngeal sample (reverse transcription polymerase chain reaction). The patient received hydroxychloroquine and azithromycin following the local Coronavirus Disease 2019 (COVID-19) treatment protocol.

**Fig 1:**
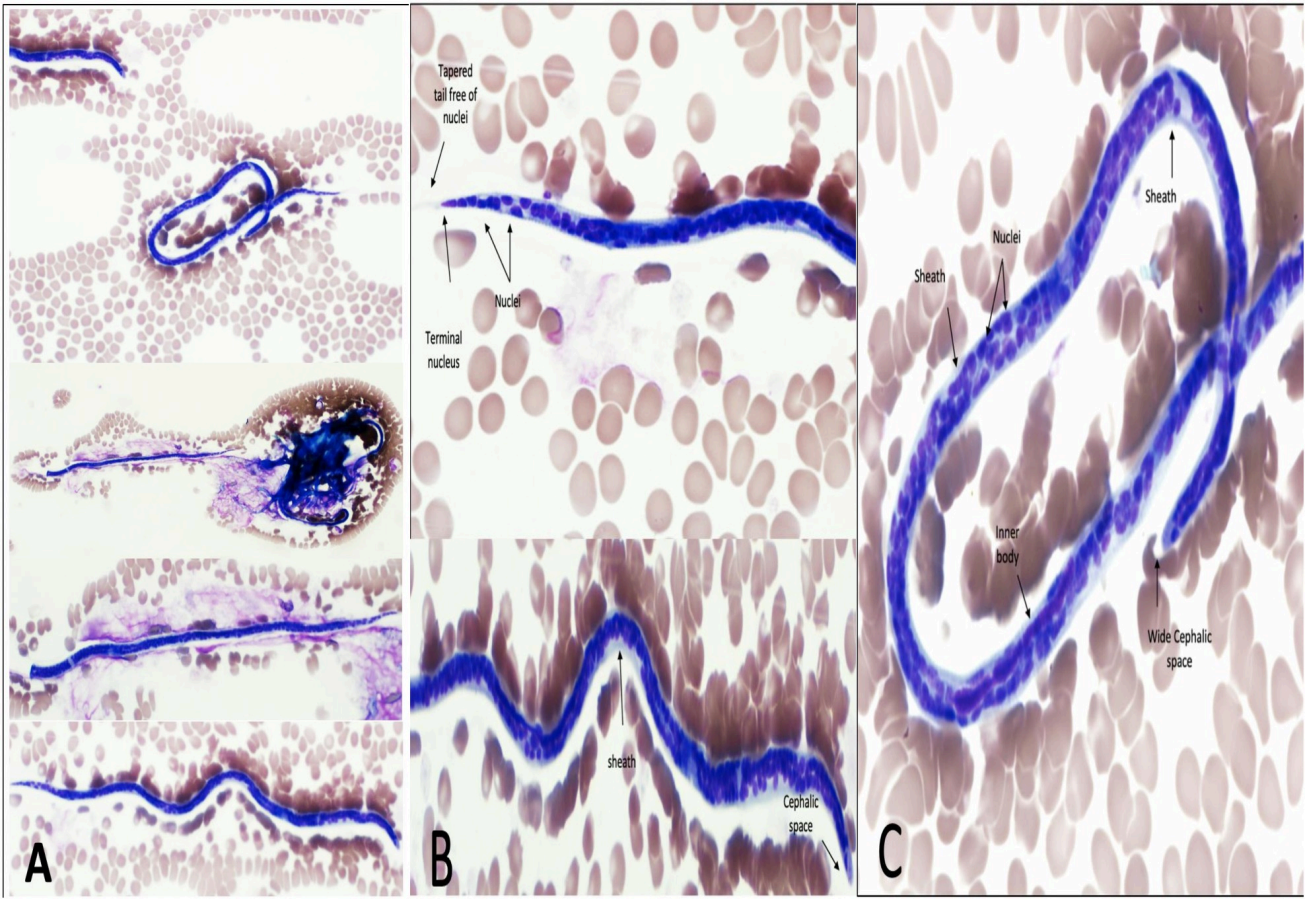
Microphotographs of *Wuchereria bancrofti* microfilaria in a peripheral blood smear. Thin blood film Giemsa stain, 100×. (A) Full-size microfilaria × 10 (B) head, body, and tail details. (C) Body and tail details.

Five days later, a blood sample revealed a microorganism’s presence. The blood smear exam confirmed the presence of *W*. *bancrofti* microfilaria (“microfilaremia level was not reported as it is not a standard test in our institute”) (Fig 1A–1C). Further guided history revealed no chronic fever, limbs or genitalia swelling, skin lesions, nocturnal cough, wheezing, dyspnea, joint pain, change in urine color, weight loss, or anorexia. The latest travel to his home country was 15 months before the presentation. A repeated focused examination did not reveal organomegaly, lymphadenopathy, hydrocele, or limb swelling.

**Table 1:** The patient’s laboratory tests.

Laboratory test	Value	Normal range
**WBC**	6.2 × 10^3^/uL	4.0–10.0^3^/uL
**RBC**	6.0 × 10^6^/uL	4.5–5.5^6^/uL
**HB**	17.7 gm/dL	13.0–17.0 gm/dL
**Platelets**	133 × 10^3^/uL	150–400^3^/uL
**Monocyte**	0.4 × 10^3^/uL	0.2–1.0^3^/uL
**Eosinophil**	0.0 × 10^3^/uL	0.0–0.5^3^/uL
**Urea**	3.8 mmol/L	2.8–8.1 mmol/L
**Creatinine**	86 umol/L	62–106 umol/L
**Sodium**	134 mmol/L	136–145 mmol/L
**Potassium**	3.9 mmol/L	3.5–5.1 mmol/L
**Chloride**	93 mmol/L	98–107 mmol/L
**Bicarbonate**	25 mmol/L	22–29 mmol/L
**Bilirubin T**	8 umol/L	0–21 umol/L
**Total protein**	86 gm/L	66–87 gm/L
**ALT**	23 U/L	0–41 U/L
**AST**	26 U/L	0–40 U/L
**CRP**	15.9 mg/L	<5 mg/L
**COVID-19 PCR**	Positive	

ALT, alanine aminotransferase; AST, aspartate aminotransferase; COVID-19, Coronavirus Disease 2019; CRP, C-reactive protein; HB, hemoglobin; PCR, polymerase chain reaction; RBC, red blood cell; WBC, white blood cell.

The patient received a single 400-mg dose of diethylcarbamazine and a 6-week oral course of 100-mg doxycycline twice daily. The hospital stay was uneventful, and 3 months after discharge, the patient remains well with no active complaints.

### Case discussion

Filariasis remains a health concern in endemic regions [5,6]. The infection is mostly asymptomatic. Alternatively, it may manifest as filarial fever, lymphangitis, arthritis, tropical pulmonary eosinophilia, or elephantiasis (limbs and genitalia swelling). Chest pain was rarely reported with filariasis. It may indicate pleural effusion or pneumothorax, and both were not present in our case [7].

The gold standard diagnosis method is worms detection in the peripheral blood smear and other tissues [2,3]. Nonetheless, as microfilariae periodically present in the blood with the peak number detected nocturnally (10 PM to 2 AM), the diagnosis can be overlooked (our patient had several prior visits and blood sampling) [8]. Rapid antigen detection assays allow infection detection at any time; thus, they have become the current standard diagnosis method [9].

Filariasis was incidentally detected in patients with various conditions. This included routine blood exam during pregnancy, axillary lymph nodes exam while staging breast cancer, and in samples of leukemia patients [10–12]. Filaria coinfection with malaria and leprosy was also reported [13,14].

COVID-19 has revealed undiagnosed chronic diseases such as DM, HTN, and chronic kidney diseases. Moreover, it unveiled the presence of chronic coexisting infections such as the human immunodeficiency virus, aspergillosis, candida, and tuberculosis [15,16]. It is known that timely identification and filariasis treatment will likely help prevent the disease’s long-term sequelae.

The filaria–COVID-19 coexistence was reported only by our case. However, this association may be underdiagnosed, particularly in endemic areas.

The immunological interaction of both COVID-19 and filariasis is complex. On the one hand, filaria escapes immune surveillance and survive in the human body for prolonged periods via antigen-specific T cell hypoactivity [17]. A muted T helper 1 response with the expansion of T helper 2 and an increase in interleukin (IL)-10 (the cytokines synthesis inhibitor) are characteristic. In order for filariasis phenotype to manifest (such as hydrocele, lymphedema, and elephantiasis), a CD4+ T cell response is typically triggered, and phenotype absence suggests T cell hyporesponsiveness. On the other hand, COVID-19 includes dysregulation of various pro-inflammatory cytokines such as the type I interferon pathway, activation of the humoral immune pathway, and increase secretion of IL-6. This dysregulated immune response is thought to contribute to a severe form of COVID-19 [18,19]. In our case, the absence of filariasis phenotypical presentation suggests a state of T cell hypoactivation. In turn, this T cell hypoactivation may have resulted in a relatively milder COVID-19 course.

An interesting finding in our case was the normal eosinophil count, unlike the majority of filariasis cases [20]. A possible explanation of this might be COVID-19 induced eosinopenia, which was reported in over 80% of COVID-19 patients [21].

As the pandemic progresses, we will likely see many comorbidities coming to the clinician’s awareness. Guided by their individual significance, clinicians will have to determine when further investigations or treatments are needed. It is prudent to keep a broad differential diagnosis in endemic areas when faced with COVID-19 patients; this is especially true when the presentation is atypical. A thorough history and physical exams are necessary to detect concomitant infections. Blanket screening for other pathogens is likely to be cost prohibitive and of low yield given the low incidence of other concomitant infections and should be discouraged until the availability of data supporting such screening.

## Conclusions

We presented the first case of filaria and SARS-CoV-2 coinfection. Clinicians in endemic areas should keep a broad differential diagnosis when faced with atypical cases of suspected COVID-19.

## Ethics statement

The Hamad Medical Corporation Institutional Review Board approval (MRC-04-20-453) was obtained for the publication of this paper. Due to the COVID-19 pandemic and its implications on direct contact with the patient, only verbal consent was obtained from the patient for the publication of this case.
